# Investigation of Microstructure and Mechanical Properties of SLM-Fabricated AlSi10Mg Alloy Post-Processed Using Equal Channel Angular Pressing (ECAP)

**DOI:** 10.3390/ma15227940

**Published:** 2022-11-10

**Authors:** Przemysław Snopiński, Augustine Nana Sekyi Appiah, Ondrej Hilšer, Michal Kotoul

**Affiliations:** 1 Department of Engineering Materials and Biomaterials, Silesian University of Technology, 18A Konarskiego Street, 44-100 Gliwice, Poland; 2 Materials Research Laboratory, Silesian University of Technology, 18A Konarskiego Street, 44-100 Gliwice, Poland; 3 Faculty of Mechanical Engineering, VSB-TU Ostrava, 17. listopadu 2172/15, 708 00 Ostrava, Czech Republic; 4 Institute of Solid Mechanics, Mechatronics and Biomechanics, Brno University of Technology, Technická 2896/2, 616 69 Brno, Czech Republic

**Keywords:** AlSi10Mg alloy, equal channel angular pressing (ECAP), microstructure, microhardness, yield strength, scanning electron microscopy, electron backscatter diffraction (EBSD)

## Abstract

With the aim of improving the excellent mechanical properties of the SLM-produced AlSi10Mg alloy, this research focuses on post-processing using ECAP (Equal Channel Angular Pressing). In our article, two different post-processing strategies were investigated: (1) low-temperature annealing (LTA) and subsequent ECAP processing at 150 °C; (2) no heat treatment and subsequent ECAP processing at 350 °C, 400 °C and 450 °C. The microstructure and mechanical properties of this alloy were analyzed at each stage of post-treatment. Metallographic observations, combined with SEM and EBSD studies, showed that the alloys produced by SLM have a unique cellular microstructure consisting of Si networks surrounding the Al-based matrix phase. Low-temperature annealing (LTA), followed by ECAP treatment, facilitated the microstructural evolution of the alloy with partial breakup of the Si network and observed nucleation of β-Si precipitates throughout the Al matrix. This resulted in a Vickers microhardness of 153 HV and a yield strength of 415 MPa. The main results show that post-processing of SLM-produced AlSi10Mg alloys using ECAP significantly affects the microstructural evolution and mechanical properties of the alloy.

## 1. Introduction

Approaches to the fabrication of parts and components made of metals and metal alloys have taken different forms in recent years. The most popular approach to metal parts forming, owing to increasing demand and productivity, has been metal additive manufacturing (AM). AM technology allows the fabrication of metallic components using metal powders or wire filaments as starting materials. Different approaches to AM, as reported in the literature, include ultrasonic additive manufacturing (UAM) [1], binder jetting [2], material jetting [3], directed energy deposition (DED) [4], powder bed fusion (PBF) [5], vat photopolymerization (VP) [6], etc. With the reported success of the PBF approach in creating efficient parts for both polymeric and metallic materials [7,8,9], it has become the most widely adopted AM technology for advanced functional materials processing. PBF uses a heat source from either a laser or an electron beam to form the desired part by melting and joining the powders of the material.

PBF technology has been subcategorized into techniques including direct laser metal sintering (DLMS) [10], selective laser sintering (SLS) [11], electron beam melting (EBM) [12], selective heat sintering (SHS) [13], and selective laser melting (SLM) [14]. SLM uses a laser beam to melt and join the atomized metallic powders uniformly distributed over a layered building platform. Due to its precision and high cooling rate (approx. 10^8^ °C/s), it is often the desired technique for building three-dimensional parts and components with soft metals, such as aluminum and its alloys. In recent years, the Al-based matrix AlSi10Mg alloy has been greatly incorporated inti industries, such as the automotive [15] and aerospace industries [16]. The mechanical properties of these alloys make them ideal for designing components that can be used in the automotive or aerospace industries.

Compared to the traditional cast, the SLM-fabricated AlSi10Mg alloy has some advantages. It has a heterogeneous microstructure composed of three major networks: a cellular network; a melt pool boundary network; and a grain boundary network. The desirable mechanical properties of this alloy, such as strain hardenability and high strength, are determined by this unique microstructure. According to the research works [17,18], a heterogeneous SLM-fabricated AlSi10Mg was found to possess a work hardening exponent of approximately 0.252, which was almost twice that of an alloy fabricated using powder metallurgy, which had a work hardening exponent of approximately 0.127. Compared to an alloy fabricated using the gravity cast method, which had a work hardening exponent of approximately 0.1, the SLM-fabricated AlSi10Mg is more desirable.

To increase the mechanical performance, the AlSi10Mg alloy is often post-processed after SLM. Considering the plastic deformation processes, the most common post-processing method is hot isostatic pressing [19]. However, this process leads to a significant reduction in strength. To improve the mechanical properties of various metals, equal channel angular pressing (ECAP) or equal channel angular extrusion (ECAE) are often used, mainly because of the refinement of microstructure and increased dislocation density [20,21,22]. For additively manufactured (AM) parts, ECAP has another advantage—it can significantly reduce their porosity [23]. However, this technique also has some disadvantages: inhomogeneity in strain distribution, complicated tooling, and high labor cost.

Although the current literature addresses post-processing approaches based on severe plastic deformation (SPD) techniques that have the potential to improve the mechanical properties of additively manufactured parts [24,25,26], a research gap exists due to the lack of studies focusing on post-processing conditions such as severe plastic deformation at elevated temperature, either in combination or not with heat treatment. Moreover, due to the novelty of the AM process compared to conventional manufacturing methods, a thorough study on the severe plastic deformation of additively manufactured products is needed.

Therefore, the objective of this research is to investigate the novel post-processing strategies for the SLM-fabricated AlSi10Mg alloy by studying the microstructure and the resulting mechanical properties. The results of this research will facilitate the development of new post-processing technologies for SLM-produced fabricated AlSi10Mg alloys to achieve the desired mechanical properties. This will attempt to shorten the post-processing time, reduce the ECAP workload, and bridge the strength/ductility trade-off of these alloys reported in the literature [22].

## 2. Materials and Methods

Gas-atomized AlSi10Mg powder produced by Sigma Aldrich was used to fabricate the samples by the selective laser melting (SLM) method. Before being used for printing, the large and sintered particles were separated by sieving the powder through a sieve with a mesh size of 63 μm.

SLM samples with dimensions 15 × 15 × 50 mm and a nearly complete density (99.92%) were fabricated using the TruPrint 1000 system (Trumpf, Ditzingen, Germany) with optimized standard parameters (laser power = 175 W; layer thickness = 20 μm; scan speed = 1400 mm/s; scan rotation = 90°; Ar atmosphere). A sample from SLM was ground to achieve the inlet dimensions of the ECAP matrix and then subjected to low-temperature annealing (LTA) at 280 °C for 9 min. This heat treatment was aimed at partially eliminating residual stresses, improving upon technological plasticity, and modifying the cellular microstructure.

The working samples were pressed once through a 90° ECAP die (introducing a strain of ε = ~1). The ECAP process was performed using samples after annealing and samples that were not annealed, at different temperatures. Table 1 lists the working samples for this work and their individual post-processing parameters.

**Figure 1:**
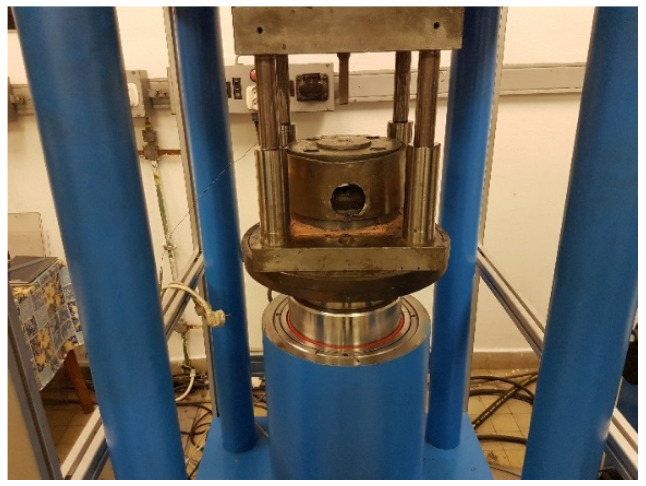
View of the workstation for material forming using the ECAP process.

The LabTest 5.2000 CT hydraulic press (Figure 1), with a maximum ram speed of 400 mm/min, was used for the ECAP experiment. In this configuration, the ram speed of the hydraulic cylinder of the press was controlled by oil pressure with the help of a servo valve and an electric motor. Heating was controlled by a dTRON 304 device equipped with a NiCr-Ni thermocouple that could be used up to a maximum temperature of 1350 °C. Since the friction effect cannot be neglected in the ECAP process, a Nicro-Thermocup 1200-type lubricant was used to reduce the friction coefficient between the ECAP sample and the ECAP die.

Metallographic samples were cut from the uniformly deformed region in the plane containing the normal direction (ND) and the transverse direction (TD) planes of the ECAP specimen for further analysis. Metallographic preparation of the specimen proceeded by grinding with SiC papers to a grit size of 1200, polishing with a coarse diamond suspension and mirror polishing with 0.04 μm colloidal silica. For EBSD analysis, mirror polishing with 0.04 μm colloidal silica was performed for one hour with an MD Chem polishing cloth to create a stress-free surface.

Microstructural analyses were conducted by a combination of optical microscopy and scanning electron microscopy (SEM) using a Zeiss Supra 35 instrument equipped with a EDAX EBSD system. Electron backscatter diffraction (EBSD) analysis was performed with an accelerating voltage of 20 kV, step size of 0.06 μm, and a tilt angle of 72°. ATEX software was used to analyze the EBSD data and to generate orientation maps, as well as to calculate the GND density based on the KAM values according to methods published elsewhere [27].

Hardness measurements (Hv) were taken over a rectangular pattern of 12 mm × 12 mm dimension (on the cross-sectional planes of the specimens) using a microhardness tester (Future-Tech FM-ARS), applying a load of 300 g for 15 s. Each indentation was made at equal intervals, separating the points at a vertical and horizontal displacement of 0.84 mm.

## 3. Results and Discussion

### 3.1. Microstructure

#### 3.1.1. Microstructure Prior to ECAP Processing

**Figure 2:**
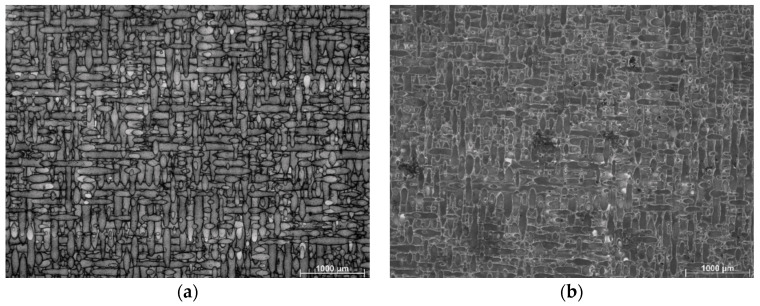
Microstructure of sample from light microscopy (**a**) SLM; (**b**) HT280.

Figure 2 shows optical micrographs of the SLM as built, as well as HT280 samples. Characteristic of the microstructure are the remaining traces of discontinuous laser scans. These traces are typically seen in SLM-fabricated metallic materials [28]. As seen in the light microscopic images, the edges of the laser scan traces are more etched, forming an outline for the scan traces. The light microscopic images do not provide a clear indication of the microstructural evolution of the LTA specimen compared to its as-built (SLM) counterpart. Therefore, a more detailed analysis with SEM is required.

**Figure 3:**
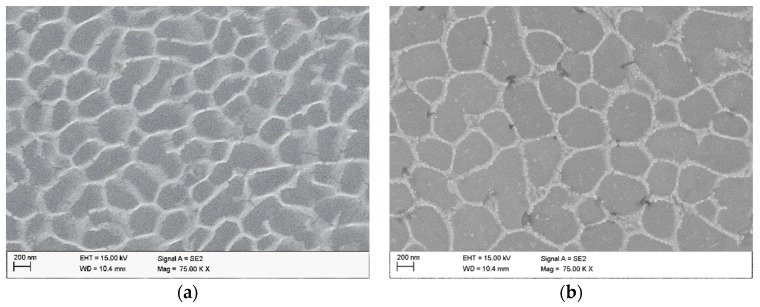
Microstructure of sample from SEM (**a**) SLM; (**b**) HT280.

**Figure 4:**
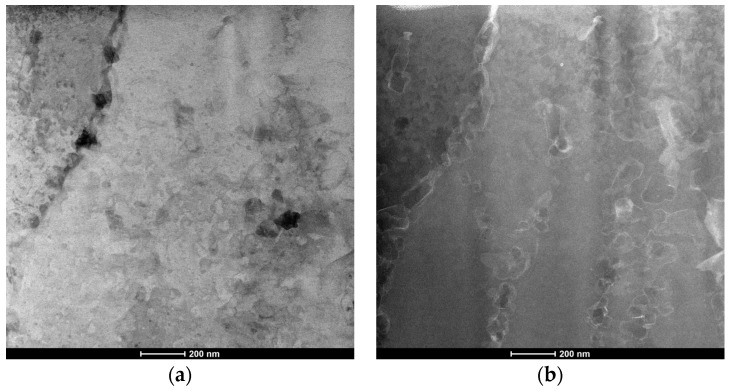
STEM (**a**) and HAADF (**b**) images of HT280 sample showing a ruptured Si network and nanosized precipitates located within aluminum cells.

Figure 3 shows the SEM images of the as-built sample (Figure 3a) and the HT280 sample (Figure 3b). A unique cellular microstructure can be seen in both samples. The cell size in the LTA sample is slightly larger than that of the SLM-fabricated (as-built) sample. The cellular structure consists of Si networks surrounding the grains of the Al matrix. As can be seen in the STEM and HAADF images (Figure 4), LTA leads to partial disintegration of the Si network around the Al matrix and precipitation of nanoscale Si precipitates which are visible inside the cellular structure.

#### 3.1.2. Microstructure after ECAP Processing

**Figure 5:**
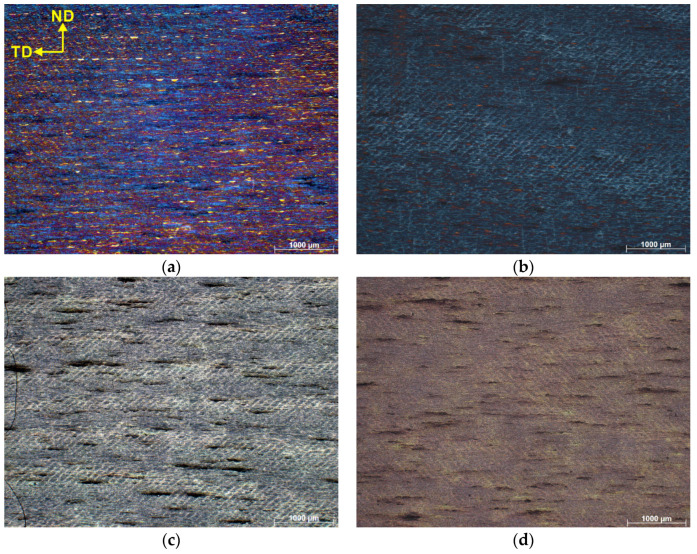
Microstructures of ECAP-processed samples (**a**) HT280E150; (**b**) SLME350; (**c**) SLME400; (**d**) SLME450.

Figure 5a shows the optical micrograph of the sample HT280E150. After chemical etching, the observed microstructure shows the so-called fish-like semicircular pattern [22], which was formed due to ECAP-induced rotation of the microstructure. After the ECAP processing of the SLM samples, Figure 5b–d, we see a similar semicircular microstructure pattern as in the HT280E150 sample. However, it should be noted that these patterns almost disappeared after ECAP processing at the highest temperature of 450 °C.

**Figure 6:**
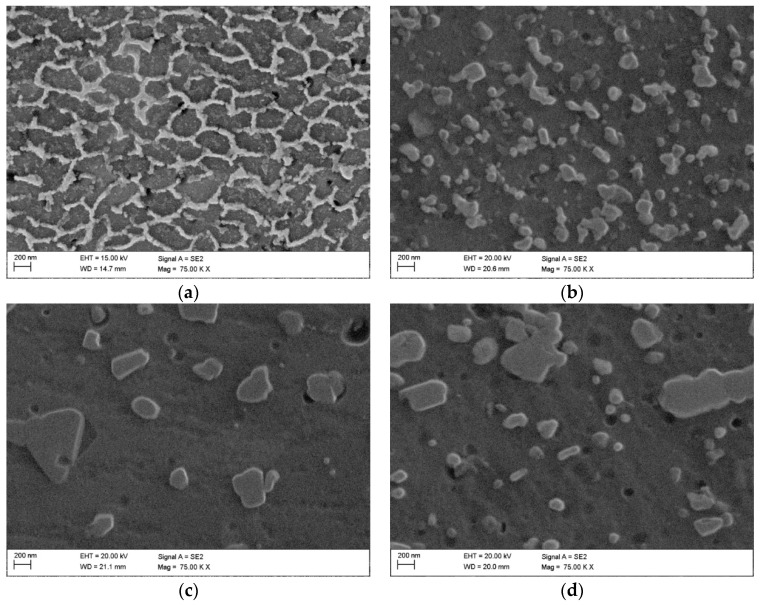
SEM images of ECAP-processed samples (**a**) HT280E150; (**b**) SLME350; (**c**) SLME400; (**d**) SLME450.

To clarify how the deformation temperature affects the cellular Si network, we acquired secondary electron images at higher magnification. Figure 6a shows the SEM image of the HT280E150 sample. It can be seen that, after ECAP pressing at 150 °C, the cellular structure shown in Figure 3b is more broken and coarser. The Si precipitates inside the cells have evolved from point-like to rod-like. In Figure 6b, we see that ECAP introduced a high dislocation, which provided a good opportunity for nucleation of precipitates on dislocation cores, resulting in complete disintegration of the cellular Si network [29].

After ECAP deformation at higher temperatures of 400 °C and 450 °C, we see the uniform distribution of large Si particles in the microstructure, see Figure 6c,d. Compared with SLME350 sample, the distances between Si particles are longer in the SLME400 and SLME450 samples, and the number of Si particles significantly decreases.

In the study [30], it is postulated that the breakup of Si networks and the formation of coarser Si particles is due to the presence of excess Si precipitates in the supersaturated Al-based matrix. It can be concluded that, after heat treatment, the supersaturated Si atoms are repelled from the Al matrix and form new small Si particles, which are initially distributed within the cellular Si network. Since the workpieces are statically annealed in the ECAP die channel during processing, there is sufficient time for the formation of coarser particles, whose size increases with increasing deformation temperature. The observed microstructural evolution of the studied samples after ECAP processing at different temperatures is in line with the observations reported by these researchers [22,27]. On the macroscale, the theoretical shear deformation pattern is in the same direction as the flow lines’ tangent, in a vertical alignment to the plane of channels intersection. Compression is therefore equally distributed in the transverse direction and the extrusion direction. On the normal direction–transverse direction (ND–TD) plane, this is seen as a parallel series of macroscopic bands. This is the most significant reason for the observation of semicircular patterns and elongated structures in the microstructure.

**Figure 7:**
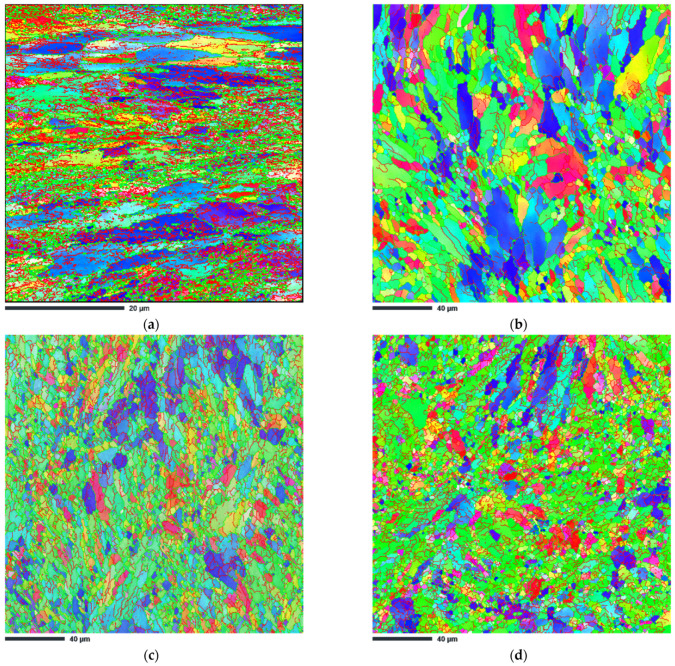
SEM IPF-Z images of ECAP-processed samples (**a**) HT280E150; (**b**) SLME350; (**c**) SLME400; (**d**) SLME450.

Electron backscatter diffraction (EBSD) analysis was performed to provide further details on the evolution of the microstructure. Figure 7 shows the inverse pole figure (IPF-Z) maps taken in the x–y plane (cross-sectional plane) for the ECAP-processed samples. In this figure, the red lines correspond to low-angle grain boundaries (LAGBs) and the green lines correspond to high-angle grain boundaries (HAGBs).

From Figure 7a and the data in Table 2, it can be seen that sample HT280E150 has an ultrafine-grained structure. The measured average grain size is 0.43 µm. For this sample, the EBSD analysis also shows a relatively high percentage of LAGBs (about 55%), indicating the formation and accumulation of multiple dislocations in the microstructure.

In contrast, the IPF-Z image of the SLME350 sample in Figure 7b shows relatively large (columnar) grains, about six times larger than those of the HT280E150 sample. In the case of this sample, the measured average grain size is about 3.3 µm. Interestingly, certain areas of fine grains can be observed in the IPF mapping along a curved region that has the exact characteristics of the melt pool (heat-affected zone). Taking into account the grain boundary (GB) misorientation, sample SLM350 is characterized by a much lower percentage of LAGBs of 37.7%, indicating recrystallization phenomena.

ECAP processing at a higher temperature of 400 °C results in more effective refinement of the microstructure. In the case of the SLME400 sample, the average grain size is about 2.1 µm, which represents a 37.4% reduction compared to the SLME350 sample (Figure 7c). However, the population of LAGBs and HAGBs remains almost the same in both samples.

ECAP processing at the highest temperature of 450 °C, as shown in Figure 7d, changes the grain structure of the alloy substantially. The boundaries of the melt pool are no longer obvious, and a large number of small grains disappear at the boundaries. Compared to the SLME400 sample, the grain size increases by 38.5% to approximately 2.9 µm. At the same time, the population of LAGBs also increases to 47.5%.

**Figure 8:**
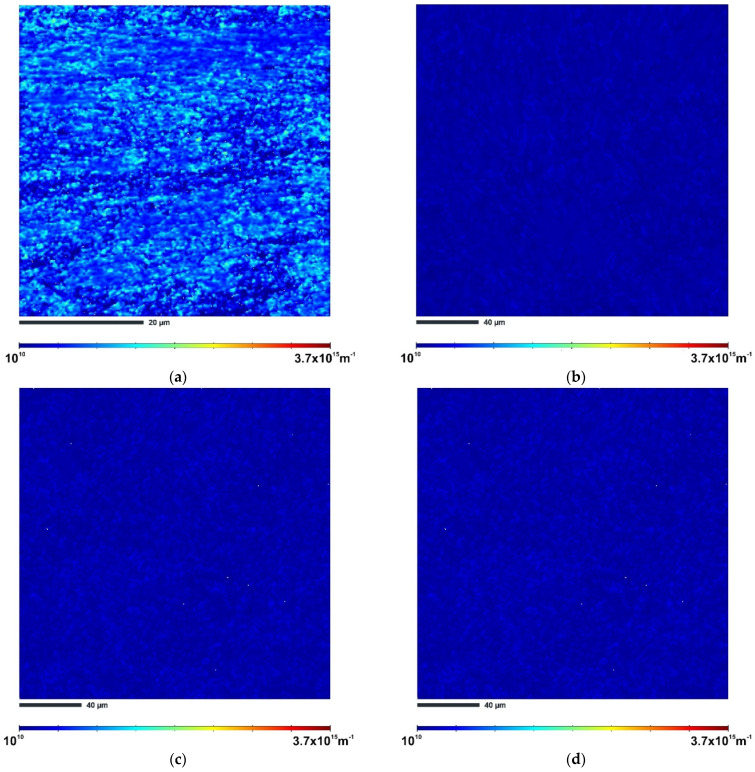
GNDs maps of ECAP-processed samples (**a**) HT280E150; (**b**) SLME350; (**c**) SLME400; (**d**) SLME450.

Maps of the geometrically necessary distribution (GND) of the samples processed via ECAP are shown in Figure 8. The lighter areas in the GND maps correspond to the areas with higher dislocation density. As can be seen, sample HT280E150 has the highest GND density of 6.70 × 10^14^ m^−2^. For the non-heat-treated samples, SLME350 exhibits a GND density of 9.60 × 10^13^ m^−2^. The SLME400 sample has a GND density of 7.69 × 10^13^ m^−2^, which is about a 20% reduction in GND density in comparison with the SLME350 sample. The GND density of the SLME450 sample is also reduced by about 11% to 6.88 × 10^13^ m^−2^ compared to the SLME400 sample, confirming that the deformation temperature as well as the initial microstructure has a significant effect on dislocation accumulation.

The formation of low-angle grain boundaries in aluminum alloys is closely related to the presence dislocations [31]. During ECAP processing, a large shear strain is introduced into the material, resulting in an overall increase in dislocation density. Since the microstructure of the SLM alloy is heterogeneous (it consists of soft Al and hard Si phases), large amounts of geometrically necessary dislocations are generated to accommodate the plastic strain gradient that develops near the Al/Si interface [32]. These GNDs move into configurations that are more energetically favorable. The result is the formation of zones with high dislocation concentrations, which appear in the form of LAGBs. In the case of sample HT280E150, the nearly full-cellular Al/Si network allowed the storage of more GNDs, resulting in the formation of multiple LAGBs. The IPF-Z and GNDs maps in Figure 7 and Figure 8 confirm the highest dislocation density in the studied areas of sample HT280E150 compared to the other ECAP-processed samples. This explains why the value of GNDs density was the highest in this sample compared to the other samples.

### 3.2. Mechanical Properties

**Figure 9:**
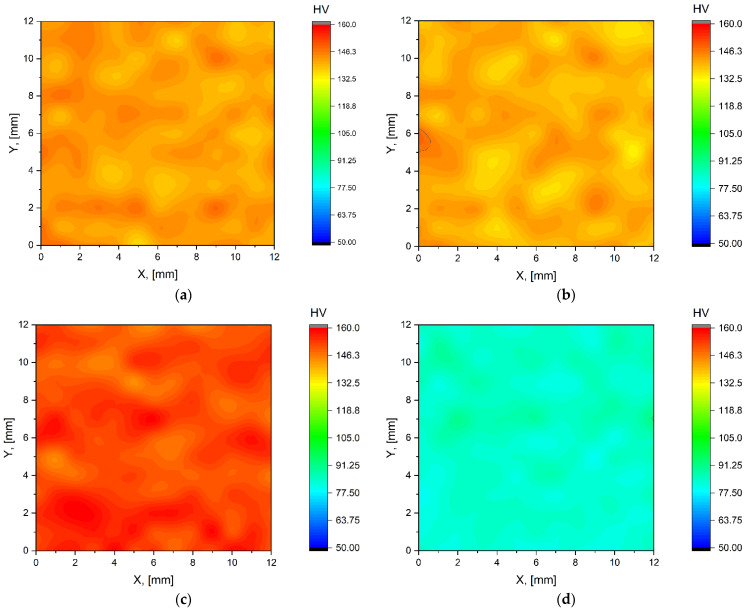

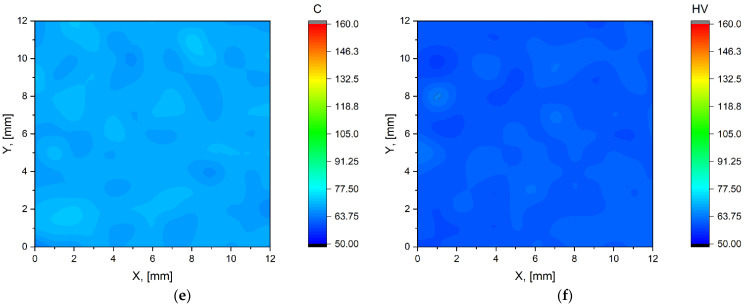
Vickers microhardness maps of studied samples (**a**) SLM; (**b**) HT280; (**c**) HT280E150; (**d**) SLME350; (**e**) SLME400; (**f**) SLME450.

The room-temperature mechanical properties of the studied AlSi10Mg samples were evaluated by Vickers microhardness and compression tests. These tests were performed on samples immediately after SLM fabrication (sample SLM), immediately after heat treatment (sample HT280), and after post-processing via ECAP. Figure 9 shows the Vickers microhardness maps. According to Figure 9a and the statistical data in Table 3, the average microhardness of the SLM sample is about 142 HV. After low-temperature annealing (HT280 sample), microhardness decreases by about 3% to 138 HV (Figure 9b) due to stress relief and partial rupture of eutectic Si network.

The heat-treated sample was pressed once through a 90° ECAP die at 150 °C, resulting in an increase in microhardness to 153 HV, which means that we achieved an improvement in microhardness of about 10% compared to the untreated samples. The microhardness value reported here for the HT280E150 sample is also higher than those published in the literature for the SLM-AlSi10Mg alloy (130–140 HV). [33,34]. Non-heat-treated samples post-processed via ECAP at higher temperatures have lower values for microhardness, as shown in Figure 9d–f. ECAP at 350 °C results in a decrease in microhardness to 86 HV, which is about 39% lower than the value of the as-built SLM sample and about 44% lower than the value of the HT280E150 sample. Further increasing the ECAP temperature to 400 °C for the SLME400 sample resulted in a 24% decrease in microhardness to 69 HV compared to the SLME350 sample. ECAP deformation at the highest temperature of 450 °C results in a further 15% decrease in microhardness to 60 HV compared to the SLME400 sample. From the metallographic and SEM observations, it can be concluded that the ECAP processing of the non-heat-treated samples (Figure 5 and Figure 6) resulted in a non-uniform microstructure of the samples. The distribution of inhomogeneity in the sample contributed to the decrease in microhardness of the samples. In contrast, for HT280E150, the stresses in the sample were relieved after heat treatment, resulting in a uniform hardness distribution during ECAP processing.

**Figure 10:**
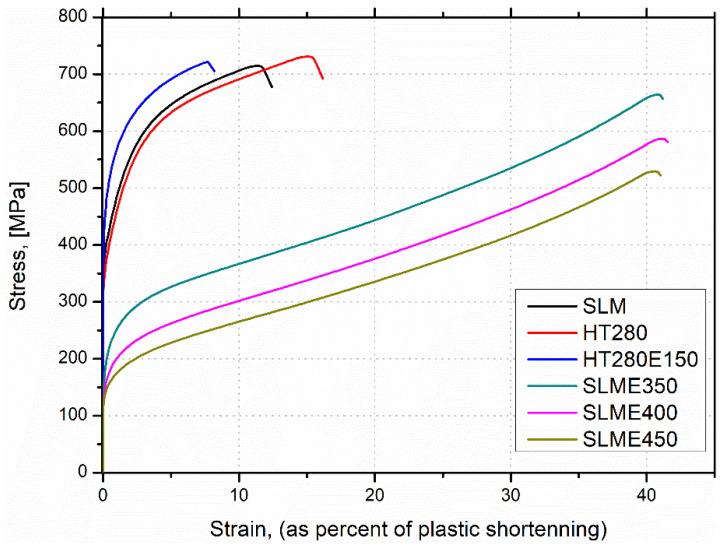
Compressive test plots of studied samples.

The yield strength results (YS) of the compression tests, Figure 10, show a pattern similar to the Vickers hardness test results. Sample HT280E150 has the highest comparative YS value of 415 MPa. This value is approximately 4% higher than the YS value of the as-built (SLM) sample, which has a YS value of 397 MPa, and about 8% higher than the heat-treated sample (HT280), which has a YS value of 385 MPa. The SLME350 sample has a YS of 187 MPa, which is about 120% lower than the HT280E150 sample. The SLME400 sample has a YS of 161 MPa, which is approximately 16% lower than the SLME350 sample. Further increasing the ECAP temperature to 450 °C results in the lowest YS value among the samples tested, which is 141 MPa, approximately 190% lower than the HT280E150 sample.

According to the mechanical property data summarized in Table 3, the HT280E150 sample exhibits the best combination of microhardness and yield strength. The superior mechanical properties of sample HT280E150 can be attributed to the unique microstructure obtained after various post-processing operations. It can be concluded that the nearly full-cellular Al/Si network in sample HT280E150 contributes more to the strength of the alloy (due to the increased storage of GND dislocations) than the fully ruptured and coarsened Si network in the SLM samples post-processed via ECAP at higher temperatures [35,36]. The grain boundary strengthening (Hall–Petch strengthening), resulting from ECAP deformation, also contributed significantly to the strength of the tested specimens, as this sample has the smallest grain size in the sub-micrometer range.

## 4. Conclusions

- In this work, the effects of ECAP processing on the microstructure and mechanical properties of SLM-fabricated AlSi10Mg alloys were studied under different conditions of post-processing heat treatment and ECAP temperatures. Resulting microstructure and mechanical properties of post-processed SLM samples were compared to that of the as-built alloy and it is observed that post-processing operations significantly affect the performance of the SLM-fabricated alloy.
- Metallographic observations, coupled with SEM investigations, revealed the SLM-fabricated alloys possessed a unique cellular microstructure made up of Si networks surrounding the Al-based matrix phase.
- Low-temperature annealing (LTA) heat treatment, followed by ECAP, processing facilitated microstructural evolution of the alloy with a partial rupture of the Si network and an observed nucleation of β-Si precipitates throughout the Al-based matrix. This resulted in a Vickers microhardness of 153 HV and a yield strength of 415 MPa, which are amongst the highest reported values in the literature for this alloy.
- Increasing the ECAP process temperature of the non-heat-treated alloys resulted in complete rupture and coarsening of the Si phase, resulting in a non-uniform hardness distribution and reducing the mechanical performance of the alloy.

## Figures and Tables

**Table 1:** Prepared samples used for the study.

Sample ID	Annealing	ECAP Temperature (°C)
HT280E150	LTA at 280 °C	150
SLME350	Not annealed	350
SLME400	Not annealed	400
SLME450	Not annealed	450
SLM	Not annealed	No ECAP
HT280	LTA at 280 °C	No ECAP

**Table 2:** Microstructural parameters obtained from EBSD analysis.

Sample	Grain Size, µm	Low Angle Boundaries, %	High Angle Boundaries, %	GNDs Density, m^−2^
HT280E150	0.44 ± 0.06	55.2 ± 1.3	44.8 ± 1.2	6.70 × 10^14^ ± 0.14
SLME350	3.37 ± 0.08	37.7 ± 1.1	62.3 ± 1.4	9.60 × 10^13^ ± 0.12
SLME400	2.11 ± 0.08	38.0 ± 1.2	62.0 ± 1.3	7.69 × 10^13^ ± 0.16
SLME450	2.91 ± 0.07	47.5 ± 1.5	52.5 ± 1.3	6.88 × 10^13^ ± 0.20

**Table 3:** Summary of mechanical properties obtained from hardness and compression tests.

Sample	Hardness, HV	Yield Strength, MPa
SLM	142 ± 2.2	397 ± 3.0
HT280	138 ± 1.8	385 ± 2.7
HT280E150	153 ± 2.5	415 ± 3.2
SLME350	86 ± 1.4	187 ± 2.4
SLME400	69 ± 2.1	161 ± 2.7
SLME450	60 ± 2.3	141 ± 2.1

## Data Availability

Data sharing not applicable.
